# Functional analysis of *Parabacteroides distasonis* F4: a novel probiotic strain linked to calf growth and rumen fermentation

**DOI:** 10.1186/s40104-025-01182-0

**Published:** 2025-04-04

**Authors:** Xiaoran Feng, Yunlong Liu, Shengyang Xu, Junnan Ma, Hao Yuan, Haixin Wang, Jiachen Hu, Sijie Jin, Shanji Liu, Jin Zhong, Tao Ma, Yan Tu

**Affiliations:** 1 Beijing Key Laboratory for Dairy Cow Nutrition, Institute of Feed Research, Chinese Academy of Agricultural Sciences, Beijing, 100081 China; 2 State Key Laboratory of Microbial Resources, Institute of Microbiology, Chinese Academy of Sciences, Beijing, 100101 China

**Keywords:** Pan-genome, *Parabacteroidesdistasonis*, Probiotic, Rumen fermentation

## Abstract

**Background:**

Rumen microorganisms are key regulators of ruminant growth and production performance. Identifying probiotic candidates through microbial culturomics presents a promising strategy for improving ruminant production performance. Our previous study identified significant differences in rumen microbial communities of Holstein calves with varying average daily gain (ADG). This study aims to identify a target strain based on the findings from multi-omics analysis and literature review, isolating and evaluating the target microbial strains from both the rumen and hindgut contents for their probiotic potential.

**Results:**

*Parabacteroides distasonis*, a strain closely associated with ADG, was successfully isolated from calf rumen content cultured with Fastidious Anaerobe Agar (FAA) medium and named *Parabacteroides distasonis* F4. Whole-genome sequencing and pan-genome analysis showed that *P. distasonis* F4 possesses a core functional potential for carbohydrate and amino acid metabolism, with the ability to produce propionate, acetate, and lactate. The results of targeted and untargeted metabolomics further validated the organic acid production and metabolic pathways of *P. distasonis* F4. An in vitro simulated rumen fermentation test showed that supplementation with *P. distasonis* F4 significantly altered rumen microbial community structure and increased the molar proportions of propionate and butyrate in the rumen. Furthermore, an in vivo study demonstrated that dietary supplementation with *P. distasonis* F4 significantly increased the ADG of pre-weaning calves.

**Conclusions:**

This study represents the first isolation of *P. distasonis* F4 from rumen, highlighting its potential as a probiotic strain for improving rumen development and growth performance in ruminants.

**Supplementary Information:**

The online version contains supplementary material available at 10.1186/s40104-025-01182-0.

## Background

The growth and development of calves directly impact the productive performance of lactating dairy cows. In particular, the average daily gain (ADG) of pre-weaning calves is positively correlated with fertility and milk yield during the lactation period [1]. Research indicates that each 100 g/d increase in calf ADG can boost first-lactation milk yield by approximately 155 kg [2]. Thus, enhancing ADG may serve as a strategic regulatory mechanism that could improve dairy productivity. Increasing research indicates that ruminal microbes are closely associated with host phenotypes [3–5]; studies suggest that around 20% of variation in feed efficiency traits such as average daily feed intake, ADG, and gain-to-feed ratio may be attributable to the composition of the rumen microbiome in beef cattle [6]. As an integral component of the rumen, the microbial community engages in interactions and competition for survival, collectively sustaining the functional stability of both the host's digestive and immune systems, thereby facilitating normal growth and development [7, 8]. The end products of ruminal microbial fermentation, volatile fatty acids (VFAs), are absorbed through the rumen epithelium by the host, promoting rumen development and providing essential energy for animals [9]. Given the crucial role of the rumen microbiome in calf ADG, manipulating these microbial populations could directly enhance growth performance and subsequent dairy productivity.

Our previous research identified microbial differences between high-ADG (HADG) and low-ADG (LADG) calves in both the rumen and hindgut. Changes in these microbial populations substantially influence fermentative processes and host metabolism, impacting ADG in calves [10]. Previous studies have also identified disparities in microbial communities and metabolites linked to different phenotypes, including feed efficiency and methane emissions. These potential biomarkers play significant roles in regulating animal epigenetic performance [11–13]. This highlights the potential for manipulating microbiomes to enhance animal productivity. Therefore, microorganisms with specific functions and uncharacterized probiotic potential hold considerable developmental value. However, current research primarily focuses on obtaining differential microbial information through multi-omics analysis [14], often lacks the isolation of specific beneficial strains, functional characterization, and elucidation of mechanisms.

In this study, building on our previous multi-omics analysis of microbial differences in HADG and LADG calves, we identified the functional bacterium *P. distasonis* with probiotic potential and successfully isolated and cultured it from the rumen fluid of calves. Using whole-genome and pan-genome analysis, we explored the potential functional characteristics and metabolites of *P. distasonis* F4 at the genetic level. We then verified its metabolic pathways through in vitro simulated rumen fermentation experiments and further validated its effects by feeding it to pre-weaning calves. A comprehensive evaluation was conducted on the potential of *P. distasonis* F4 as a probiotic strain to enhance the ADG of calves.

## Materials and methods

### Functional probiotic extraction

**Fig. 1:**
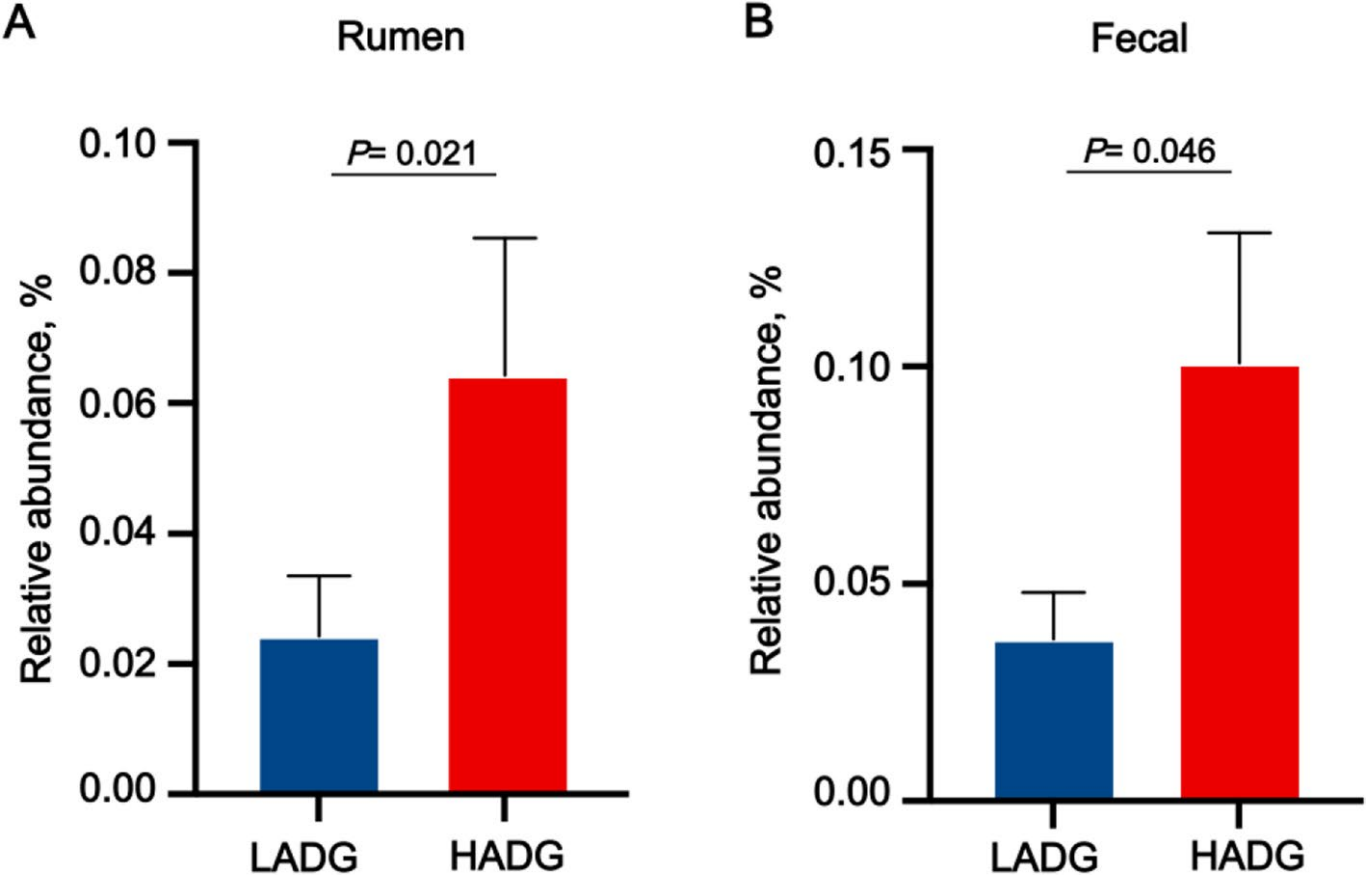
Comparison of *P. distasonis* abundance between HADG and LADG groups in the rumen (**A**) and feces (**B**)*.* The vertical axis represents the relative abundance of *P. distasonis*. HADG, higher average daily gain group; LADG, lower average daily gain group

The differences in rumen microbial community structure between calves with HADG and LADG were compared, and differentially abundant microbial biomarkers were identified using Linear Discriminant Analysis Effect Size (LEfSe) in our previous study [10]. Additionally, we applied the same method to analyze species-level differences in the fecal microbiota. After identifying the microbial biomarkers, we conducted an extensive literature review to evaluate their culturability and basic biological characteristics [15–20], ultimately selecting *P. distasonis* as our candidate functional probiotic strain (Fig. 1).

### Strain screening and identification

The strain isolation experiment utilized healthy calves as the host source, with the primary objective of isolating target microbial strains from the rumen contents of these calves. The experiment was conducted at the Beijing Key Laboratory for Dairy Cow Nutrition (Beijing, China). Rumen contents of Holstein calves were collected using a rumen tube at Beijing Sunlon Livestock Development Co., Ltd. (Changping District, Beijing, China). The rumen contents were filtered through four layers of gauze, promptly placed into a 50-mL centrifuge tube pre-filled carbon dioxide (CO_2_), and then transferred to an anaerobic chamber with an ice pack (0–4 °C) for transportation back to the laboratory. The strain screening was carried out by gradient dilution and plate coating at the Microbial Culture Platform of the Institute of Microbiology, Chinese Academy of Sciences (Beijing, China). The anaerobic workstation was maintained under the following specific parameters: a temperature of 37 °C, 0% oxygen (O_2_), 3.8% CO_2_, 96.2% nitrogen, and a relative humidity of 74% (RH). The procedure was followed: the sample was continuously diluted in 10-fold steps with PBS, and 100 μL of each dilution (10^−3^, 10^−4^, and 10^−5^) was plated onto solid media. The plates were cultured under anaerobic conditions for 48 h. A total of seven different solid media were used during the screening process (Additional file 1: Table S1). After 48 h of cultivation, individual colonies were carefully selected and subjected to a purification process involving at least three generations of streaking to ensure the isolation of definitive strains.

The purified strains were identified by Sanger sequencing to obtain the bacteria’s 16S rDNA at RuiBiotech Co., Ltd. (Beijing, China). The total bacterial DNA was extracted and purified using a bacterial genome DNA extraction kit (Tiangen Biochemical Technology, Beijing, China). The 16S rDNA was amplified by PCR using universal bacterial primers 27F (5'-AGAGTTTGATCCTGGCTCAG-3') and 1492R (5'-ACGGCTACCTTGTTACGACT-3'). The PCR reaction mixture consisted of 15 μL 2 × EasyTaq SuperMix, 1.2 μL of each primer (10 μmol/L), 6 μL template DNA, and 6.6 μL ddH_2_O, with a final volume of 30 μL. The PCR amplification conditions were as follows: 94 °C for 5 min, followed by 35 cycles of 94 °C for 30 s, 56 °C for 30 s, 72 °C for 45 s, with a final extension at 72 °C for 10 min, and storage at 4 °C. Sequencing was performed by RuiBiotech Co., Ltd. (Beijing, China) using the Sanger method with an ABI 3730xl sequencer. The closest relatives of the isolated strains were determined through a sequence similarity search in public databases, and sequences of the closest relatives were retrieved from GenBank. A phylogenetic tree was constructed using Neighbor-Joining method with MEGA 6.0 software to identify the bacterial species.

### Complete genome sequencing

Genomic DNA of this strain was extracted using a Bacterial DNA extraction kit (Majorbio, shanghai, China) according to the manufacturer’s protocol. The purified genomic DNA was quantified, and high-quality DNA was selected for further research. Genome sequencing was performed using a combination of PacBio Sequel IIe and Illumina sequencing platforms. The sequencing data generated from both platforms were used for subsequent bioinformatics analyses. All analyses were conducted using the free online platform of Majorbio Cloud Platform (http://cloud.majorbio.com) provided by Shanghai Majorbio Bio-pharm Technology Co., Ltd. The detailed procedures are as follows. The raw Illumina sequencing reads generated from the paired-end library were quality-filtered using Fastp v0.23.0 [21]. The HiFi reads generated from the PacBio platform were used for further analysis. The clean short reads and HiFi reads were assembled to construct complete genomes using Unicycle v0.4.8 [22] and Pilon v1.22 (https://github.com/broadinstitute/pilon/) was used to polish the assembly using short-read alignments to reduce small error rates. The coding sequences (CDSs) of the chromosome and plasmid were predicted using Glimmer [23], tRNA-scan-SE (v2.0) [24] was used for tRNA prediction, and Barrnap v0.9 (https://github.com/tseemann/barrnap) was used for rRNA prediction. The predicted CDSs were annotated using the Clusters of Orthologous Groups of Proteins (COG) and KEGG databases through sequence alignment tools such as DIAMOND v0.8.35 (https://github.com/bbuchfink/diamond) and HMMER v3.1b2 (http://hmmer.org/), with default parameters.

### Construction of the pangenome

A total of 1,099 whole-genome sequences of *P. distasonis* were obtained from the NCBI Genome Database. These sequences were screened based on geographic location, isolation source, host, genome size, contig N50, contig L50, and genome coverage. Ensuring sufficient diversity and sequence quality, 85 whole-genome sequences along with the *P. distasonis* F4 genome (F4) that were isolated and cultured in the present study were selected to construct the pangenome. A detailed list of these accessions is presented in the Supplementary Materials (Additional file 1: Table S3). The genomes were annotated using Prokka (v1.14.6) [25], resulting in annotation files in GFF format. Based on the GFF files, Roary (v3.13.0) [26] was used for pan-genome analysis. Based on the presence and absence of genes among different genomes, core genes (99% ≤ genomes ≤ 100%), soft core genes (95% ≤ genomes < 99%), shell genes (15% ≤ genomes < 95%), and cloud genes (0% ≤ genomes < 15%) were defined. Using the gene information, the core gene sequences and F4 strain specific gene sequences were extracted from pangenome reference.fa. Functional annotation of the core genes and strain-specific genes was then performed using EggNOG-mapper (v2.1.12) [27].

### Metabolic characterization of *P. distasonis* F4

*P. distasonis* F4 was cultured in Fastidious Anaerobe Broth (FAB) for 48 h, followed by centrifugation to obtain the supernatant for untargeted metabolomics analysis. The concentration of VFAs was measured using gas chromatography (Agilent 7890B) [28]. LC-MS analyses were conducted using a Vanquish UHPLC system (Thermo Fisher Scientific, USA) at Suzhou PANOMIX Biomedical Tech Co., Ltd. (China). The MSConvert tool within the Proteowizard package (v3.0.8789) [29] was used to convert the raw mass spectrometry data into mzXML format. Peak detection, filtering, and alignment were carried out using the RXCMS software package, with support vector regression correction applied based on quality control (QC) samples to eliminate systematic errors; only metabolites with a coefficient of variance (CV) of less than 30% in QC samples were retained for subsequent analysis. Metabolites identification involved referencing HMDB (http://www.hmdb.ca), MassBank (https://massbank.jp), KEGG (https://www.genome.jp/kegg/), LIPID MAPS (http://www.lipidmaps.org), mzCloud (https://www.mzcloud.org), and a proprietary metabolite database constructed by Suzhou PANOMIX Biomedical Tech. Co., Ltd.

### Basic characteristics of *P. distasonis* F4

#### Growth characteristics

The strain was activated on FAA solid medium, and single colonies were inoculated into FAB for anaerobic incubation at 37 °C for 12 h. The culture was then transferred to fresh FAA at a 1% dose and incubated anaerobically at 37 °C. Optical density (OD) at 600 nm was measured every 2 h using a microplate reader, with vortexing before each measurement. The growth curve was plotted using time on the *x*-axis and OD values on the *y*-axis.

#### Tolerance testing

Acid resistance: FAB was adjusted to pH 3, 4, 5, and 7, sterilized, and inoculated with 3% of the bacterial solution. The pH 7 medium served as the control. After 12 h at 37 °C, the cultures were plated and bacterial survival rates at each pH were calculated.

Bile salt tolerance: FAB containing 0.15%, 0.3%, and 0.6% (w/v) cow bile salt was prepared. The bacterial solution was inoculated at 3%, with standard FAB as control. Cultures were incubated anaerobically at 37 °C for 12 h, after which bacterial counts were taken.

Artificial gastric and intestinal fluid tolerance: the bacterial solution was inoculated at 3% into artificial gastric juice (pH 3.0, FAB with 1 mg/mL pepsin) and artificial intestinal juice (pH 8.0, FAB with 1 mg/mL trypsin). Cultures were incubated anaerobically at 37 °C for 4 h, with standard FAB as the control.

Antimicrobial susceptibility: the agar disk diffusion (ADD) method was used to assess susceptibility, with *Escherichia coli* as the control. After evenly spreading the bacterial suspension on FAA plates, drug-sensitive paper discs were placed and incubated anaerobically at 37 °C for 24 h. Clear zone diameters were measured, and results were evaluated according to Clinical and Laboratory Standards Institute (CLSI) guidelines.

###  In vitro fermentation

#### Treatment and sample collection

This study strictly complied with the requirements of the Animal Ethics Committee of the Chinese Academy of Agricultural Sciences (Beijing, China); the approval number is IFR-CAAS20231015. Two substrates were used in this experiment: milk replacer and a 57.9% milk replacer + 42.1% starter mix. The nutrient composition of the milk replacer and starter are present in Table S5. Each substrate contained six fermenters with two treatments: Control (30 mL blank medium) and Microbe (30 mL of *P. distasonis* F4 at 10^9^ CFU/mL), with three replicates each. Rumen fluid was collected from fistulated Holstein cattle, filtered, and transferred to the laboratory in pre-heated sealed containers. For fermentation, 10 g of substrate was mixed with 300 mL artificial rumen culture fluid (prepared using rumen fluid and buffer at a 1:2 ratio) and incubated in an AMPTS II system for 48 h. The rumen buffer was prepared according to Menke [30]. Prior to fermentation initiation, the fermentation bottles were purged with CO_2_ for 2–3 min to establish an anaerobic environment by displacing residual O_2_. After fermentation, pH was measured, and 10 mL of liquid was stored at −20 °C for VFA analysis. Additionally, 2 mL was stored at −80 °C for microbial analysis.

#### DNA extraction and 16S rRNA genes sequencing

Genomic DNA was extracted using the E.Z.N.A.® DNA kit (Omega Bio-tek, Norcross, GA, USA) and the quality and concentration were assessed via NanoDrop2000 (Thermo Scientific, USA). PCR amplification was performed on the V3–V4 variable region of the 16S rRNA gene using primers 338F (5'-ACTCCTACGGGAGGCAGCAG-3') and 806R (5'-GGACTACHVGGGTWTCTAAT-3') with barcode sequences. The amplification conditions were: 95 °C for 3 min, followed by 27 cycles of 95 °C for 30 s, 55 °C for 30 s, and 72 °C for 30 s, with a final extension at 72 °C for 10 min. Sequencing was conducted using the Illumina PE300 platform (Shanghai Majorbio Bio-pharm Technology Co., Ltd.). Paired-end sequencing data were quality-controlled using Fastp (v0.19.6), and the sequences were merged using FLASH (v1.2.11) [31]. The DADA2 [32] plugin in QIIME2 [33] was used to denoise the sequences and generate amplicon sequence variant (ASV) tables. Representative reads of each ASV were selected using the QIIME2 package.

### Fluorescent quantitative PCR

Total genomic DNA from ruminal fluid was extracted by E.Z.N.A.®DNA kit (Omega Bio-tek, Norcross, GA, USA), and genomic DNA of *P. distasonis* F4 was extracted using a bacterial genomic DNA extraction kit. The specific primers were synthesized by Shengong Bioengineering Co., Ltd. (Shanghai, China). Primer sequences were F (5'-TCATCGTTTACTGCGTGGACTACC-3') and R (5'-AGCCTGCCAAGCCATGACTG-3'). After confirming primer specificity, the amplified fragment was ligated to the pMD18-T vector, resulting in the construction of a recombinant plasmid as the standard product. Quantitative PCR (qPCR) SYBR Green Master Mix (Novozan, Nanjing, China) was utilized for fluorescence quantitative determination using a Bio-Rad qPCR instrument (USA). The qPCR conditions were set as follows: 95 °C for 3 min, 95 °C for 5 s, 58 °C for 30 s,72 °C for 1 min, for 40 cycles. Following establishment of the standard curve, absolute quantification of bacterial samples was performed.

### Animal, diet, and experimental design

This experiment was conducted at Luan County Shounong New Oasis Modern Pasture Co., Ltd. (Tangshan, China) with protocols approved by the Animal Ethics Committee of the Institute of Feed Research of Chinese Academy of Agricultural Sciences (approval number: IFR-CAAS20240429). Forty newborn Holstein calves were collected and weighed within 2 h of birth, and randomly assigned to two groups based on body weight (BW). Each group included 20 replicates with 8 male and 12 female calves. The control group (CON) received normal saline (20 mL/d/herd), while the treatment group (PDH) was supplemented with *P. distasonis* F4 (10^9^ CFU/mL, 20 mL/d/herd). All treatments were administered once daily prior to morning feeding. All calves received 4 L of colostrum within the first 2 h after birth and were housed individually in single hutches. Milk was provided twice daily at the following amounts: 7 L/d (2–7 d), 9 L/d (8–34 d), 12 L/d (35–56 d), and 8 L/d (57–63 d); from 64 to 70 days of age, milk was provided once daily at 4 L. Clean water was available ad libitum. Starter was offered ad libitum from 3 to 70 days of age; the compositions and nutrient levels are detailed in Table 1. Body weight of calves at birth, at 35 days of age, and at weaning were recorded, along with daily milk and starter intake, to facilitate the calculation of ADG, dry matter intake (DMI), and feed conversion ratio (FCR).

**Table 1:** Composition and nutrient level of calf diet (DM basis), %

Item	Diets	
	Starter	Milk
Ingredient		
Corn	23.94	
Soybean meal	21.54	
Rapeseed meal	6.00	
Wheat bran	4.00	
Wheat flour	5.00	
DDGS^1^	8.00	
Corn gluten feed (sprayed)	1.42	
Expanded soybeans	1.00	
Rolled corn	8.00	
Sugarcane molasses	5.00	
Mold inhibitor	0.10	
Whey powder	10.00	
Premix^2^	4.00	
Nutrient levels		
Gross energy, MJ/kg	17.13	
Dry matter (fresh basis)	95.89	11.70
Ash	7.75	-
Crude protein	24.26	28.80
Ether extract	4.91	36.75
Neutral detergent fiber	21.61	-
Acid detergent fiber	13.93	-
Calcium	1.36	-
Phosphorus	0.59	-

^1^*DDGS* Distillers dried grains with solubles

^2^Premix provides per kg of feed: VA 12,000 IU, VD 2,200 IU, VE 20 mg, Fe 60 mg, Cu 12.5 mg, Mn 50 mg, Zn 24 mg, Se 0.3 mg, I 1.0 mg, Co 0.3 mg

### Statistical analysis

Statistical analyses of in vitro fermentation parameters, ADG, DMI and FCR were performed using the independent sample *t*-test in SPSS Statistics software (v25.0, IBM Corp., USA), with a significance level of *P* < 0.05. Principal coordinate analysis (PCoA) based on the Bray-Curtis distance algorithm was used to assess microbial community structure similarities among samples, with PERMANOVA employed to evaluate the significance of community structure differences. LEfSe analysis (LDA > 2, *P* < 0.05) was used to identify significantly different bacterial groups from the phylum to genus level between groups. Most of the statistical visualizations were performed using R studio (v2023.06.2+561).

## Results

### Bacterial identification and complete genome sequence

**Fig. 2:**
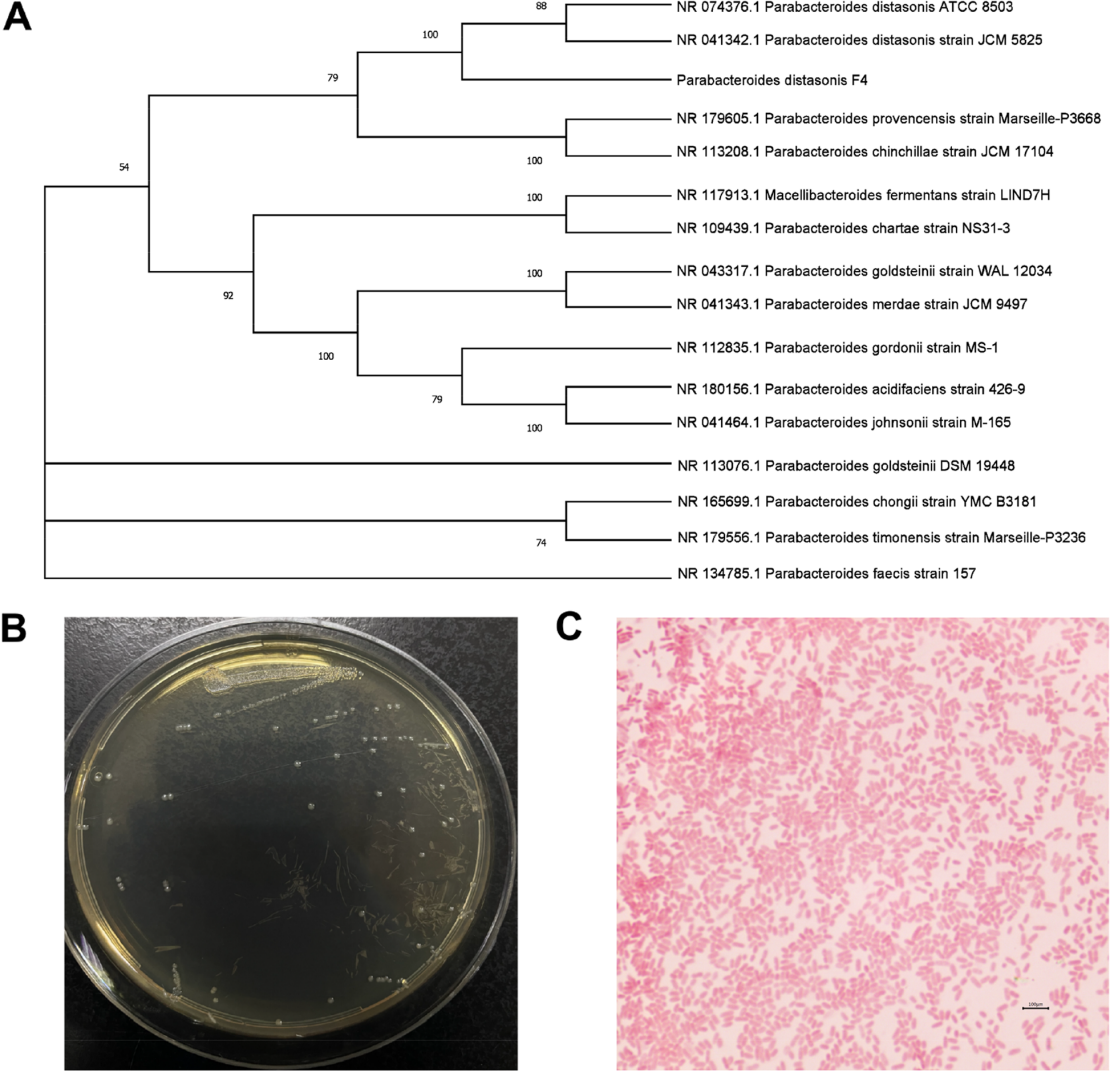
Phylogenetic tree based on 16S rDNA sequences and colony morphology of *P. distasonis* F4. **A** The evolutionary history was inferred using the Neighbor-Joining method. The bootstrap consensus tree, based on 1,000 replicates, represents the evolutionary history of the taxa analyzed. **B** Colony morphology on the FAA plate: colonies are gray-white, smooth, with neat edges and translucent. **C** Microscopic examination at 400× magnification shows red, short rod-shaped bacteria, classified as Gram-negative

The target strains were successfully isolated on FAA. The nearly complete 16S rDNA sequence (Additional file 1: Table S2) identified the F4 strain as *P. distasonis*. This strain was identified as a new strain and named *P. distasonis* F4. A phylogenetic tree was constructed (Fig. 2A), illustrating the phylogenetic location of the F4 strain. Colony morphology and microscopic examination results of *P. distasonis* F4 are shown in Fig. 2B.

**Fig. 3:**
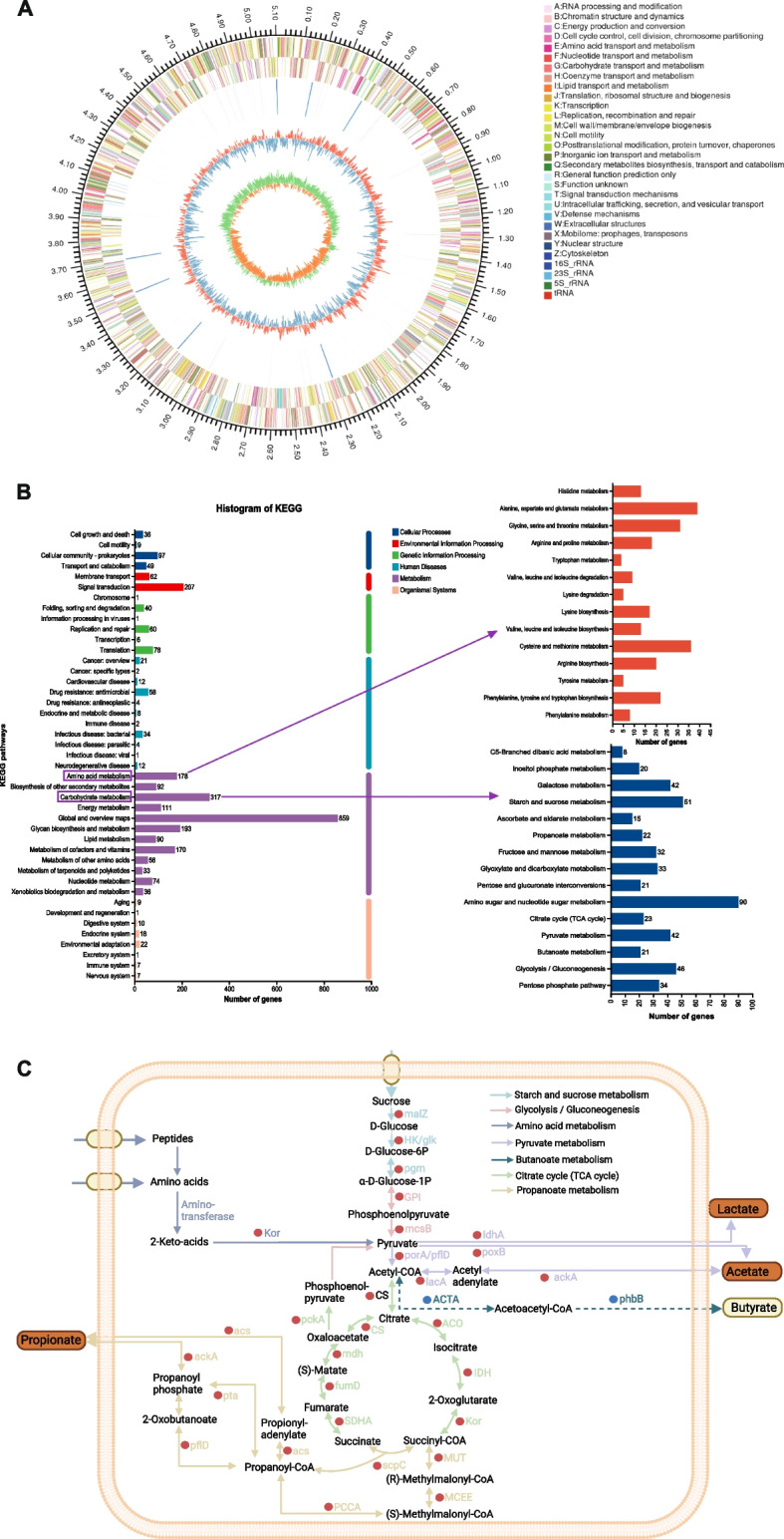
The genome Circos and KEGG functional prediction for *P. distasonis* F4. **A** The Circos plot represents the genome structure. The outermost circle indicates the genome size. The second and third circles represent the CDSs on the positive and negative strands, respectively, with different colors denoting different COG functional classifications of the CDSs. The fourth circle marks rRNA and tRNA. The fifth circle depicts GC content, with red sections indicating regions with GC content above the genome-wide average (higher peaks indicate greater deviation), and blue sections indicating regions below the average (higher peaks indicate greater deviation). The innermost circle represents the GC-Skew value, calculated as (G − C)/(G + C). **B** The left side shows KEGG Level 2 pathway classification, with the *y*-axis representing the Level 2 categories of KEGG pathways and the *x*-axis indicating the number of genes annotated under each category. The right side provides a detailed exhibition of the Level 3 classifications within the major metabolic pathways of carbohydrate metabolism and amino acid metabolism. **C** Predicted pathways for acid production are shown. Red circles represent present genes, blue circles represent absent genes, and dashed lines indicate blocked pathways

The genome of *P. distasonis* F4 consists 5,119,619 bp in length, with a G+C content of 45.12%. Genome analysis revealed 4,263 predicted CDSs, spanning 4,635,489 bp, with a mean length of 1,087 bp. These CDSs occupied 90.54% of the total genome. The genome harbored 21 rRNA genes, comprising 7 copies each of 5S, 16S, and 23S rRNA operons. Furthermore, 82 tRNA genes and 133 repetitive elements were identified, including 44 scattered repeats and 89 tandem repeats (Fig. 3A).

The KEGG annotation indicated that metabolic pathway-related genes represented the largest portion of the genome (71.53%), with most genes associated with global and overview maps (859 genes), carbohydrate metabolism (317 genes), glycan biosynthesis and metabolism (193 genes), and amino acid metabolism (178 genes). Further analysis highlighted the involvement of key pathways, including starch and sucrose metabolism, propanoate metabolism, the citrate cycle (TCA cycle), pentose phosphate pathway, glycolysis/gluconeogenesis, and amino acid metabolism (alanine, aspartate, glutamate, cysteine, and methionine) (Fig. 3B). The acid production pathway of *P. distasonis* F4 is depicted in Fig. 3C. Functional analysis of the genome through COG revealed 3,377 functional genes, categorized into 24 groups. The most abundant categories included cell wall/membrane/envelope biogenesis (390 genes), carbohydrate transport and metabolism (287 genes), and inorganic ion transport and metabolism (243 genes) (Additional file 2: Fig. S1).

### Pan-genome analysis of *P. distasonis*

**Fig. 4:**
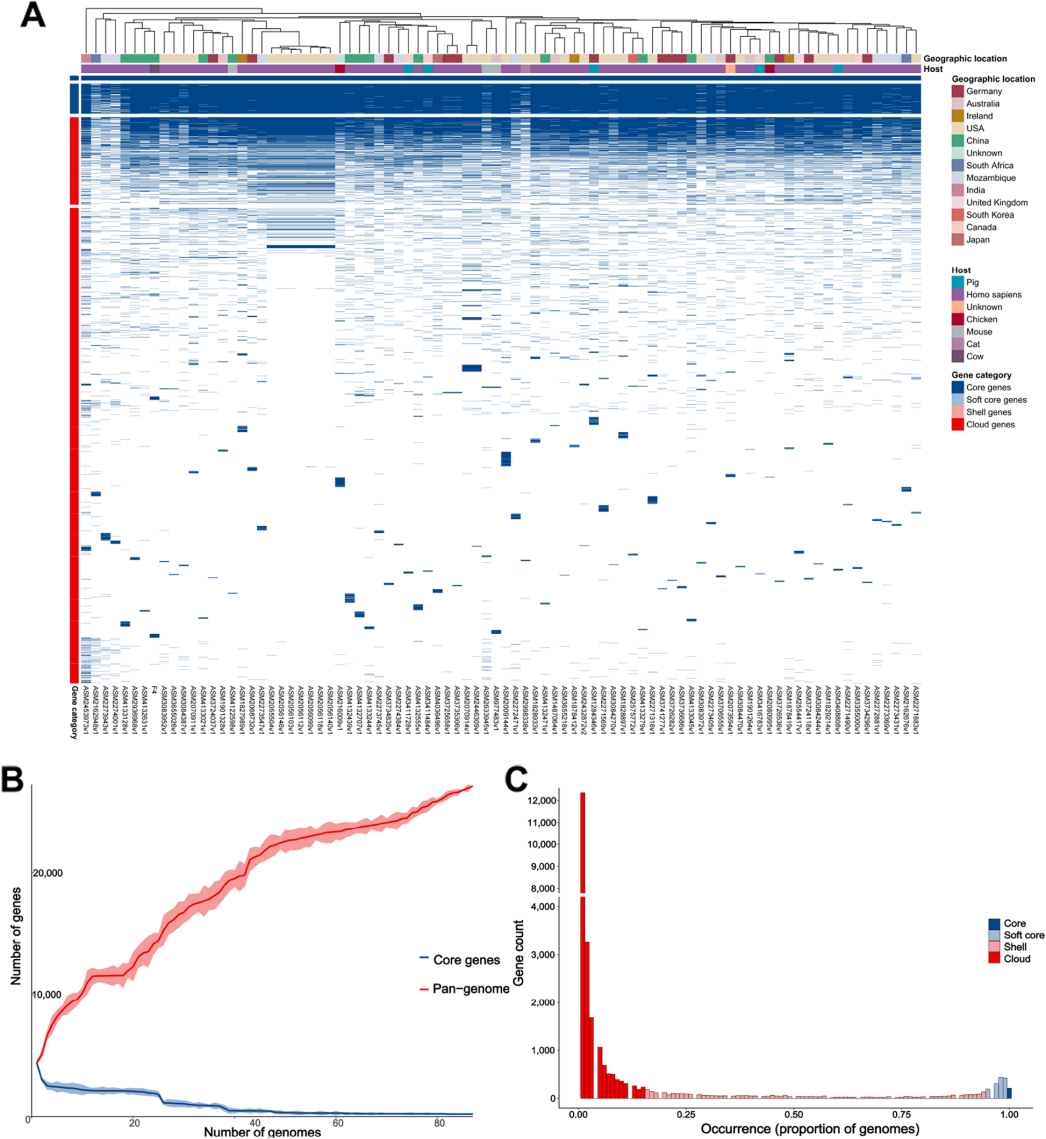
The pan-genome analysis characteristics of *P. distasonis.* **A** The heatmap displays the gene expression profiles of *P. distasonis* sourced from different countries and hosts, with each column representing a different genome sample; **B** The rarefaction curves show an increase in the pan-genome size (red line) and decrease in the core genes size (blue line). **C** The frequency of core, soft core, shell, and cloud genes plotted against the number of genomes

To comprehensively investigate the *P. distasonis* genome, we analyzed the pan-genomes of 85 fully assembled *P. distasonis* genomes from diverse geographical regions and hosts (Fig. 4A). Most strains were isolated from humans, with *P. distasonis* F4 being the only strain derived from cattle, providing novel insights into strain specificity and increasing strain resource diversity. The pan-genome analysis identified 213 core genes, 1,357 soft core genes, 3,758 shell genes, and 21,802 cloud genes, out of a total of 27,130 genes. Furthermore, the pangenome of *P. distasonis* exhibited an addition of approximately 181 genes when the 85^th^ genome was included, highlighting the “open” nature of the *P. distasonis* pan-genome. In contrast, the core genome showed a sharp decline with the addition of early genomes, stabilized after the inclusion of the first 83 genomes, suggesting a closed core genome. Additionally, individual gene frequencies were assessed, and a cumulative gene distribution map was generated (Fig. 4B). The high proportion of cloud genes underscores significant heterogeneity among the examined *P. distasonis* strains and further emphasizing the open nature of pangenome (Fig. 4C**)**. A comprehensive KEGG functional of the 213 core genes revealed a prominent (Additional file 1: Table S4**)** associated with carbohydrate metabolism. Additionally, these genes contribute significantly to amino acid metabolism, as well as other essential metabolic pathways, including the metabolism of cofactors and vitamins, energy metabolism, and various other processes.

### Metabolic characteristics of *P. distasonis* F4

**Fig. 5:**
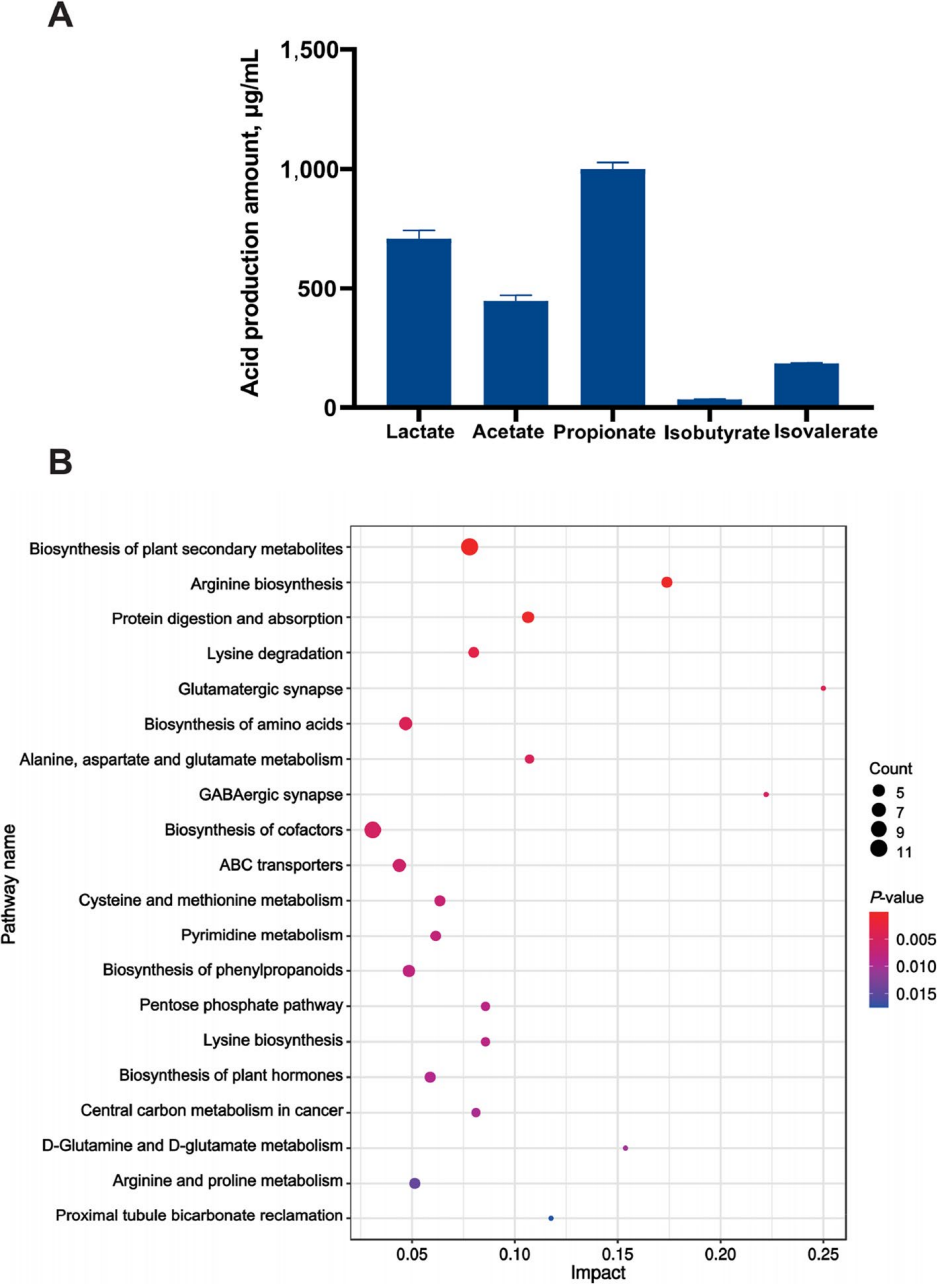
The metabolic characteristics of *P. distasonis* F4. **A** The acidogenic capability of *P. distasonis* F4. **B** The horizontal axis represents the Impact values enriched within different metabolic pathways, while the vertical axis signifies the enriched pathways themselves. The size of the points is proportional to the number of metabolites corresponding to each pathway. The color coding is associated with the *P*-value, where a more intense red color indicates a lower *P*-value, thus suggesting greater statistical significance, and a bluer color implies a higher *P*-value

Gas chromatography analysis showed that *P. distasonis* F4 produces lactate, acetate, and propionate, with propionate being the most abundant product (Fig. 5A). Enrichment analysis further highlighted the involvement of *P. distasonis* F4 in carbohydrate and amino acid metabolism pathways. Specifically, pathways such as arginine biosynthesis, arginine and proline metabolism, alanine, aspartate and glutamate metabolism, cysteine and methionine metabolism, lysine degradation, lysine biosynthesis, and the pentose phosphate pathway were significantly enriched (*P* < 0.05; Fig. 5B).

### Potential probiotic properties of *P. distasonis* F4

**Fig. 6:**
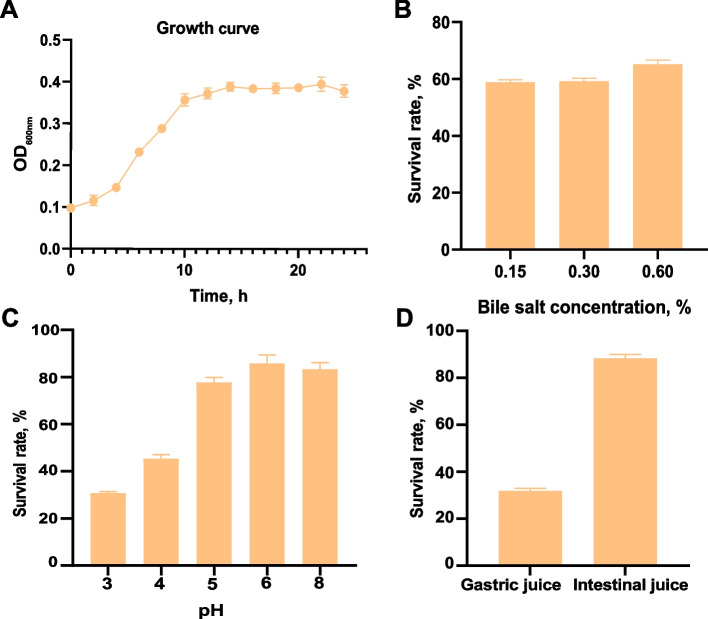
The in vitro cultivation characteristics of *P. distasonis* F4*.* **A** The growth curve observed after 24 hours of cultivation. **B** The survival rate under varying bile salt concentrations. **C** The acid and alkali tolerance of *P. distasonis* F4. **D** The growth performance of *P. distasonis* F4 in artificial gastric and intestinal juice

The growth curve of *P. distasonis* F4 in FAB (Fig. 6A) showed a lag phase from 0 to 2 h, followed by logarithmic growth from 2 to 14 h, and reaching a stable phase at 16 h. The maximum viable bacterial count was 4.21×10^9^ CFU/mL. The strain maintained a survival rate of over 30% in a liquid medium at pH 3 and demonstrated tolerance to varying concentrations of bile salts. Furthermore, survival rates of 31.74% in artificial gastric juice and 88.32% in artificial intestinal juice were observed (Fig. 6B–D). All tests conducted using the plate colony counting method showed viable bacteria counts exceeding 10^6^ CFU/mL, meeting the criteria for the addition of probiotics. The antimicrobial susceptibility of *P. distasonis* F4 varied significantly across different agents. The strain was highly susceptible to chloramphenicol, tetracycline, polymyxin, and vancomycin, with moderate susceptibility to norfloxacin. In contrast, resistance was observed against ampicillin, cefazolin, amikacin, gentamicin, and cotrimoxazole, as no inhibition zones were detected in these tests (Table 2).

**Table 2:** Antibiotic resistance of *P. distasonis* F4 to ten antimicrobial agents

Antimicrobial agent	Standard diameter of drug sensitive ring, mm			Diameter of drug susceptibility ring, mm	Susceptibility
	R	I	S		
Ampicillin	≤13		≥17	-	R
Cefazolin	≤14	15–17	≥18	-	R
Amikacin	≤14	15–16	≥17	-	R
Gentamicin	≤12	13–14	≥15	-	R
Cotrimoxazole	≤10	11–15	≥16	-	R
Norfloxacin	≤12	13–16	≥17	15.0	I
Chloramphenicol	≤12	13–17	≥18	31.2	S
Tetracycline	≤14	15–18	≥19	21.6	S
Polymyxin	≤8	9–11	≥12	16.7	S
Vancomycin	≤8	9–12	≥13	14.6	S

R: Drug resistance, I: Neutral sensitivity, S: Sensitivity, -: No inhibition zone

### In vitro fermentation parameters and microbial colonization

When milk replacer was used as the substrate, the molar proportion of propionate in Microbe group was significantly higher than that in the Control group (*P* < 0.05). In addition, the ratio of acetate to propionate and the molar proportion of valerate was significantly lower in the Microbe group compared to the Control group (*P* < 0.05). There were no significant differences in total volatile fatty acids (TVFA), acetate, isobutyrate, and isovalerate between the two groups (*P* > 0.05). In fermentation with a mixture of milk replacer and starter, the Microbe group significantly increased the molar proportion of butyrate and decreased the molar proportion of isovalerate (*P* < 0.05; Table 3). No significant differences were observed in gas production or pH between the groups (*P* > 0.05; Additional file 1: Table S6).

**Table 3:** Effects of *P. distasonis* F4 on rumen fermentation parameters of in vitro fermentation

Substrates^1^	Treatment^2^	TVFA, mmol/L	Molar proportion, mmol/100 mmol						
			Acetate	Propionate	Isobutyrate	Butyrate	Isovalerate	Valerate	A/P^3^
Milk replacer	Control	187.07	42.42	28.01^b^	1.42	15.28	2.72	10.15^a^	1.52^a^
	Microbe	185.94	40.31	30.22^a^	2.35	15.70	2.54	8.87^b^	1.33^b^
	SEM	10.618	0.884	0.299	0.631	0.263	0.244	0.308	0.045
	*P*-value	0.920	0.075	0.013	0.213	0.181	0.498	0.014	0.048
Mixed	Control	182.21	44.75	29.86	0.81	13.35^b^	1.87^b^	9.36	1.50
	Microbe	182.41	43.91	29.80	1.21	13.49^a^	2.08^a^	9.50	1.47
	SEM	6.327	0.496	0.190	0.523	0.036	0.065	0.195	0.023
	*P*-value	0.977	0.221	0.778	0.311	0.019	0.032	0.495	0.365

^1^This experiment used two substrates: milk replacer and a 57.9% milk replacer + 42.1% starter mix

^2^Control, blank medium for substrate; Microbe, the substrate contained 10^9^CFU/mL of *P. distasonis* F4

^3^A/P, the ratio of acetate to propionate

^a,b^Data in the same rank with different superscripts differ (*P* < 0.05)

**Fig. 7:**
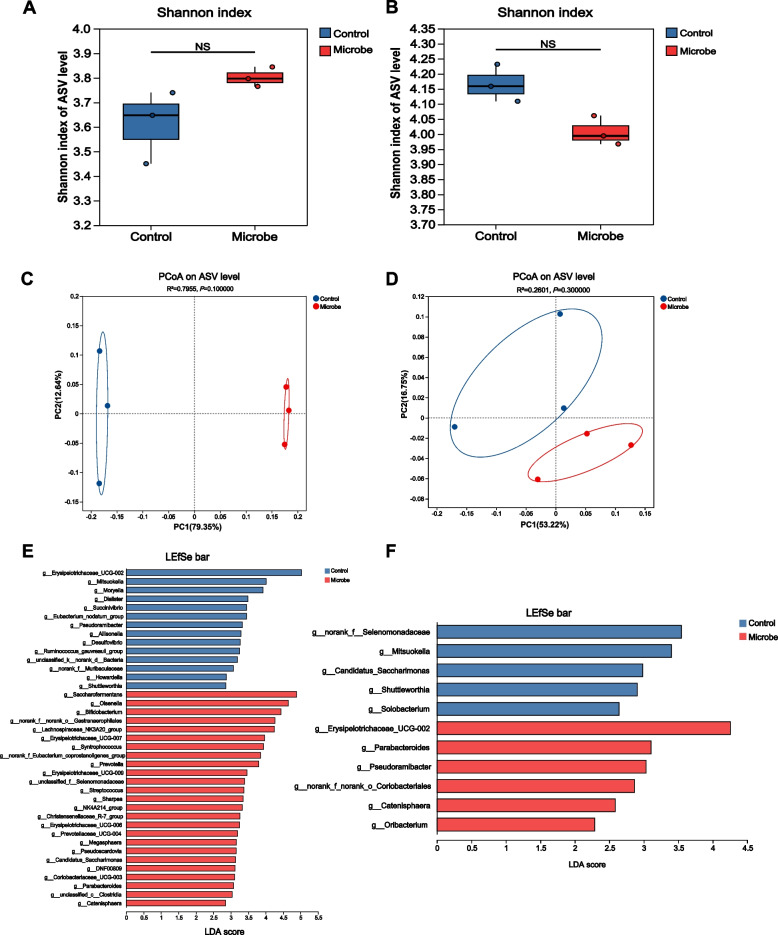
Analysis of microbial diversity and genus-level differential bacteria from in vitro fermentation experiment. **A**, **C**, **E** Substrate with milk replacer. **B**, **D**, **F** Substrate with milk replacer and starter mixture. **A** and **B** Shannon index (NS: not significant). **C** and **D** PCoA plots showing principal coordinate components (percentages indicate variance explained). **E** and **F** LEfSe analysis of differentially abundant genera (LDA score > 2, *P* < 0.05). Control, blank medium for substrate; Microbe, the substrate contained 10^9^ CFU/mL of *P. distasonis* F4

Shannon diversity analysis showed no significant differences (*P* > 0.05) between the Control and Microbe groups, regardless of whether milk replacer or a mixture of milk replacer and starter was used as the fermentation substrate (Fig. 7A and B). Bray-Curtis-based PCoA showed clear separation along the PC1 axis between the Control and Microbe groups when milk replacer was used as the substrate (Fig. 7C), indicating differences in microbial community structure. Although community differentiation was observed, statistical significance was not reached. LEfSe analysis identified several differentially abundant microbial taxa at the genus level (Fig. 7E). The Microbe group significantly increased the abundance of potential probiotics, including *Bifidobacterium*, *Lachnospiraceae_NK3A20_group*, *Christensenellaceae_R-7_group* and *Parabacteroides*, while reducing the abundance of opportunistic pathogens such as *Dialister* and *Desulfovibrio*. When using the mixture of milk replacer and starter as the substrate, PCoA also showed separation between the two groups, but there was also no statistical difference (Fig. 7D). LEfSe analysis revealed a significant increase in the abundance of *Erysipelotrichaceae_UCG-002*, *Parabacteroides*, *Pseudoramibacter*, *Catenisphaera*, and *Oribacterium* in the Microbe group at the genus level (Fig. 7F).

**Fig. 8:**
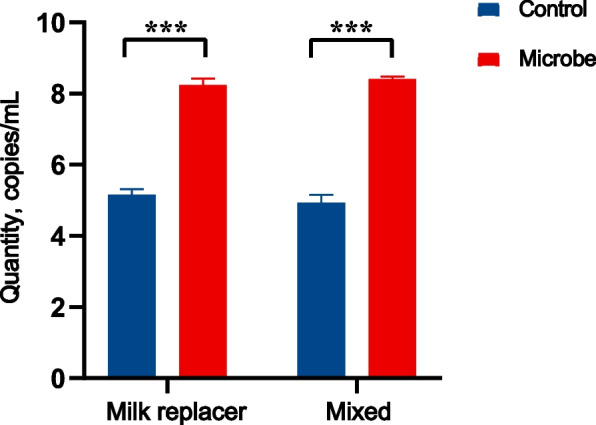
Plot of absolute quantitative results for *P. distasonis* F4. The horizontal axis represents the different substrates, the vertical axis indicates the absolute quantitative copy number. *** represents *P* < 0.01.

qPCR results indicated significantly higher bacterial DNA copy numbers of *P. distasonis* F4 in the Microbe group compared to the Control group for both the milk replacer and mixed substrate treatments (*P* < 0.01; Fig. 8). This demonstrated a substantial increase in bacterial abundance in the Microbe group for both substrates.

### Growth performance in calves

There were no significant differences in initial weight and BW at 35 d between CON and PDH groups (*P* > 0.05). However, an increasing trend emerged by the 70 d in the PDH group (0.05 < *P* < 0.10). No significant differences in DMI were observed between the groups at all periods (*P* > 0.05). The ADG and FCR were significantly higher in the PDH group than in the CON group during the 35 to 70 d period (*P* < 0.05), with an upward trend over the entire 1 to 70 d period (0.05 < *P* < 0.10) (Table 4).

**Table 4:** Effects of *P. distasonis* F4 on growth performance of calves

Items	Groups		SEM	*P*-value
	CON	PDH		
BW^1^, kg				
Initial	42.00	42.71	2.189	0.749
35 d	75.29	74.78	2.443	0.836
70 d	104.18	108.63	2.436	0.076
DMI^2^, kg				
1 to 35 d	1.07	1.08	0.013	0.954
35 to 70 d	1.60	1.60	0.840	0.986
1 to 70 d	1.33	1.33	0.047	0.981
ADG^3^, kg/d				
1 to 35 d	0.95	0.91	0.051	0.499
35 to 70 d	0.83^b^	0.97^a^	0.056	0.015
1 to 70 d	0.89	0.94	0.030	0.087
FCR^4^				
1 to 35 d	1.16	1.21	0.067	0.459
35 to 70 d	2.03^a^	1.68^b^	0.140	0.016
1 to 70 d	1.52	1.42	0.058	0.091

*CON* The control group received saline (20 mL/d), *PDH* Supplemented with *P. distasonis* F4 (10^9^ CFU/mL, 20 mL/d)

^1^*BW* Body weight

^2^*DMI* Dry matter intake, including milk and starter

^3^*ADG* Average daily gain

^4^*FCR* Feed conversion ratio, the ratio of DMI to ADG

^a,b^Data in the same row with different superscripts differ (*P* < 0.05)

## Discussion

Rumen microbiota plays an important role in influencing the growth performance of calves, as previously reported in our studies linking microbiota to ADG [10]. However, most current studies focus on identifying differential microbial communities through multi-omics analyses, with limited attention to the functional characterization and validation of specific strains. In the present study, we successfully isolated *P. distasonis* F4, a strain significantly associated with improved growth performance in calves. This Gram-negative anaerobic bacterium, belonging to the Bacteroidetes phylum [15]*,* is recognized as a core member of the human intestinal microbiome and is regulated by dietary factors [16]. Previous studies have demonstrated its therapeutic potential in various conditions, including rheumatoid arthritis, multiple sclerosis, and metabolic diseases [17–20]. Notably, *P. distasonis* has been shown to stimulate intestinal gluconeogenesis, modulate appetite, promote hepatic glycogen synthesis, and improve glucose metabolism [18]. Based on these findings, we hypothesize that *P. distasonis* derived from calves may possess probiotic potential. Therefore, we conducted a comprehensive analysis of its functional traits to validate its probiotic potential and metabolic effects.

The whole-genome sequencing and pan-genomic analysis of *P. distasonis* F4 have enhanced our understanding of its genetic composition. Our pan-genome analysis, which included 85 *P. distasonis* genomes from public databases and the F4 strain isolated from calves, confirmed that *P. distasonis* F4 is the only strain derived from calves, thus expanding the strain’s genetic resource pool. By categorizing the pan-genome into core, soft core, shell, and cloud genes, we provide insights that could facilitate genetic engineering for strain optimization. According to Heaps' law, the open nature of the *P. distasonis* pan-genome suggests that many more genes may be discovered as additional strains are analyzed [34]. Core genes comprised a small portion of the genome, while cloud genes accounted for a large share, highlighting significant genetic variability among strains from diverse geographic locations and hosts. This variability may result from mutations, environmental adaptation, or evolutionary pressures [35]. Although our analysis did not identify unique genes specifically linked to rumen digestion in strain F4, this likely reflects the complexity of the rumen environment. Nonetheless, our comparative genomic analysis provides valuable insights into the genomic diversity of *P. distasonis* and underscores the need for continued exploration of its probiotic potential [36]. Comprehensive KEGG functional annotation and metabolomic analysis showed that *P. distasonis* F4 exhibited significant capabilities in carbohydrate metabolism and amino acid metabolism. Considering our previous research that highlights the correlation between microbes and acid production, we predicted the organic acid production pathway of *P. distasonis* F4 based on annotated genes. The predicted pathways of this portion of the acid-producing pathway were nearly perfectly consistent with metabolomic results. The metabolic characteristics indicated that *P. distasonis* F4 mainly produced propionate, acetate, and lactate but lacked the ability to produce butyrate. This limitation is attributed to the absence of key enzymes, ACTA (EC: 2.3.1.9) and phbB (EC: 1.1.1.36), which are crucial for butyrate biosynthesis. Most of the annotated genes were involved in glycolysis, the TCA cycle, pyruvate metabolism, and propionate metabolism, leading to the production of propionate and lactate as key metabolites. These findings provide valuable insights into the metabolic potential of *P. distasonis* F4 and its functional role within the host environment.

To further confirm the applicability of *P. distasonis* F4, we explored its probiotic properties in vitro. An essential prerequisite for probiotics is their ability to withstand the harsh gastrointestinal (GI) environment, particularly resilience to pepsin, trypsin, and bile salts [37]. The GI system typically contains bile salt concentrations ranging from 0.3% to 0.5%, and food transit through the small intestine takes approximately 4 h [38]. Our findings demonstrate that *P. distasonis* F4 exhibited strong tolerance to simulated gastric fluid and bile salts, maintaining a viable bacterial count exceeding 10^6^ CFU/mL after exposure. This resilience meets the criteria for probiotic incorporation into formulations, highlighting the strain’s probiotic potential. In addition, due to the common use of antibiotics in livestock management, it is important to assess the antibiotic sensitivity of probiotic strains to ensure their safe application [39] Our resistance profiling showed that *P. distasonis* F4 is resistant to ampicillin, cefazolin, amikacin, gentamicin, and co-trimoxazole, while displaying varying sensitivity to norfloxacin, chloramphenicol, tetracycline, polymyxin, and vancomycin. Understanding this antibiotic profile provides practical insights for its strategic use in production settings. The in vitro assessment of *P. distasonis* F4 preliminarily proved its promising probiotic potential, positioning it as a candidate for further exploration as a novel feed additive for ruminants.

Previous studies have shown that early microbial colonization direct impact the host phenotype and plays a crucial role in shaping the rumen microbiota of young animals [40, 41]. To access the impact of *P. distasonis* F4 on rumen fermentation at different pre-weaning stages, we designed two distinct fermentation substrates based on the dietary characteristics of pre-weaned calves. It is worth noting that, the quantitative findings fully validate the successful establishment of our in vitro test model, as well as confirm successful colonization and proliferation of bacteria with added strains within the rumen environment. Using milk replacer as the substrate, the increased molar proportion of propionate and reduced the ratio of acetate to propionate indicate that *P. distasonis* F4 altered the fermentation pattern, promoting propionate production. Propionate is the only VFA capable of supporting net glucose synthesis in ruminants [42], making its increased production significant for providing more efficient energy to support growth [43]. The intricate relationship between rumen microorganisms and VFAs is tightly regulated, and changes in the microbial community directly influence VFA profiles [44]. *Christensenellaceae*_R-7_group, which has been shown to correlate with feed efficiency and propionate concentration [45], plays a key role in rumen development and nutrient absorption [46]. Similarly, *Megasphaera*, a core rumen bacterium, produces both acetate and propionate by utilizing lactic acid, contributing to the prevention of rumen acidosis [47]. Additionally, *Coriobacteriaceae UCG-003* has been positively correlated with propionate production, suggesting its role in enhancing feed efficiency [48]. LEfSe analysis demonstrated that these bacterial genera significantly increased in the Microbe group, aligning with the observed increase in propionate levels. In the group utilizing a combined substrate of milk replacer and starter, the increase in butyrate production may be attributed to enhanced microbial interactions, leading to a higher abundance of butyrate-producing bacteria. For example, *Pseudoramibacter* has been reported to produce formate, acetate, and butyrate as fermentation end-products [49]. However, the precise mechanisms underlying these metabolic pathways require further investigation to understand their contributions to rumen fermentation fully. The animal experiment further validated the regulatory impact of *P. distasonis* F4 on calf growth performance, confirming its association with increased ADG. The precise mechanisms underlying these effects warrant further investigation.

Although advances in cultivation-omics technologies have significantly improved the ability to isolate and cultivate previously unculturable strains [50], certain limitations persist. In the present study, we focused on validating the probiotic potential of *P. distasonis* F4, as it was the primary strain identified with potential significance. However, this narrowed focus leaves many other differential bacteria unexplored. As research continues to identify new potential probiotics, leveraging cultivation-omics to explore a broader range of microbial species will be crucial. This will enhance the understanding of microbial diversity and expand the repertoire of beneficial strains available for probiotic applications.

## Conclusion

In this study, we successfully isolated and cultured *P. distasonis* F4 from calves, a key strain associated with improved ADG. The core function of *P. distasonis* F4 lies in carbohydrate metabolism, particularly in the production of short-chain volatile fatty acids, providing a foundational basis for its development as a functional probiotic to enhance animal growth performance. Both in vitro and in vivo feeding trials validated its effectiveness, indicating that *P. distasonis* F4 has significant potential as a novel feed additive to promote ADG in calves.

## Supplementary Information

Additional file 1: Table S1. The information and compositions of the seven culture-medium. Table S2. The 16S rDNA sequence of *P. distasonis* F4. Table S3. 85 whole-genome sequences of *P. distasonis*. Table S4. The KEGG functional categories for 213 core genes within *P. distasonis*. Table S5. Nutrient composition of Milk replacer and stater of in vitro fermentation. Table S6. Total gas production and pH over 48 h with varying substrates and treatments.

Additional file 2: Fig. S1. Functional Annotation of COG for *P. distasonis *F4. The horizontal axis represents different COG categories, while the vertical axis represents the number of genes. For specific functional descriptions of each COG category, please refer to the legend on the right.

## Data Availability

This whole genome shotgun project has been deposited at GenBank under the accession JAYMFS000000000, and the BioProject accession is PRJNA1062702. The sequencing data during the current study are available from the NCBI Sequence Read Archive (SRA), accession number PRJNA1222191.

## Abbreviations

ADG: Average daily gain
ADD: Agar disk diffusion
ASV: Amplicon sequence variant
BW: Body weight
CDS: Coding sequence
CLSI: Clinical and laboratory standards institute
CO_2_: Carbon dioxide
DMI: Dry matter intake
COG: Clusters of orthologous groups of proteins
FAA: Fastidious Anaerobe Agar
FAB: Fastidious anaerobe broth
FCR: Feed conversion ratio
GI: Gastrointestinal
HADG: Higher average daily gain
LADG: Lower average daily gain
LEfSe: Linear Discriminant Analysis Effect Size
O_2_: Oxygen
OD: Optical density
PCoA: Principal coordinate analysis
qPCR: Quantitative PCR
TVFA: Total volatile fatty acids
