# Supplementary material for: Functional analysis of Parabacteroides distasonis F4: a novel probiotic strain linked to calf growth and rumen fermentation
Source: J Anim Sci Biotechnol. 2025 Apr 4;16:50. doi: 10.1186/s40104-025-01182-0 (PMC11969818; doi:10.1186/s40104-025-01182-0)
Supplement: Supplementary file 2 — Additional file 2: Fig. S1. Functional Annotation of COG for P. distasonis F4. The horizontal axis represents different COG categories, while the vertical axis represents the number of genes. For specific functional descriptions of each COG category, please refer to the legend on the right. [file 40104_2025_1182_MOESM2_ESM.docx]

**
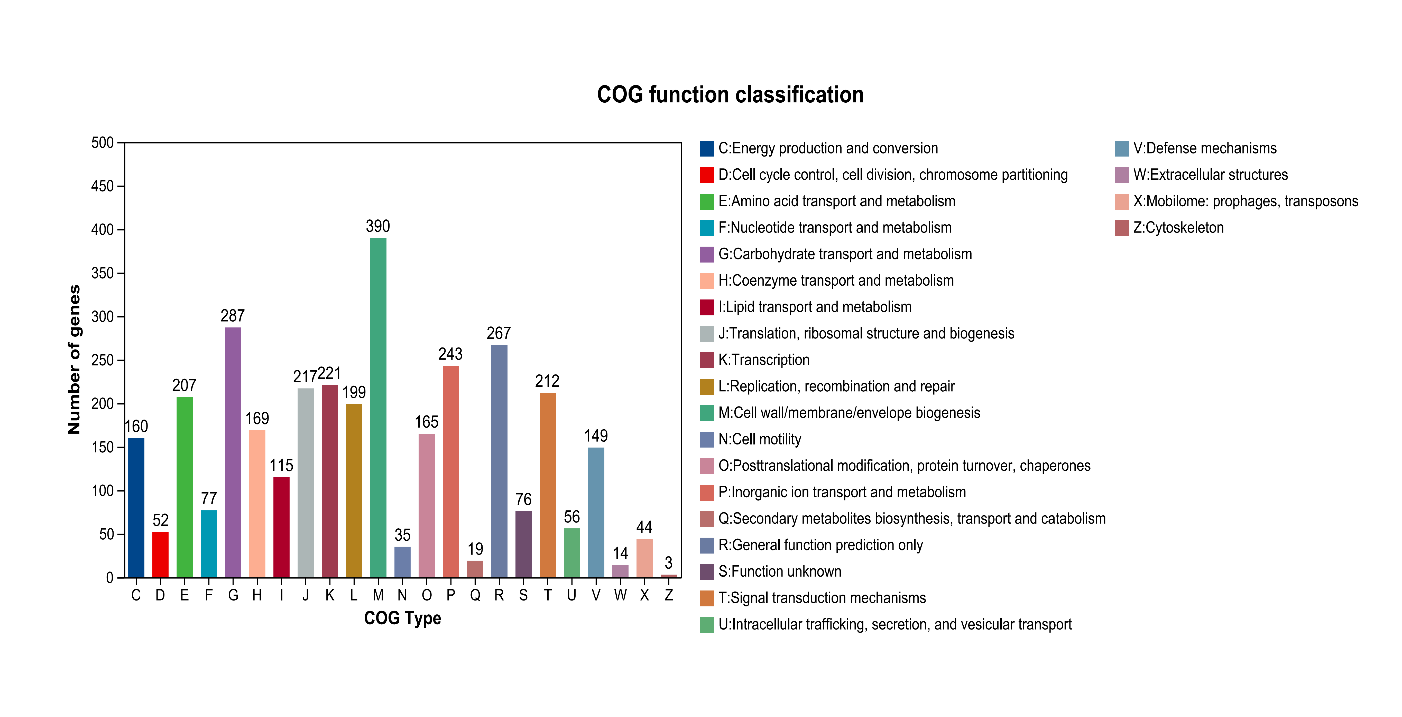
Fig. S1** Functional Annotation of COG for *P. distasonis* F4. The horizontal axis represents different COG categories, while the vertical axis represents the number of genes. For specific functional descriptions of each COG category, please refer to the legend on the right
